# Evaluation of a Biostimulant (Pepton) Based in Enzymatic Hydrolyzed Animal Protein in Comparison to Seaweed Extracts on Root Development, Vegetative Growth, Flowering, and Yield of Gold Cherry Tomatoes Grown under Low Stress Ambient Field Conditions

**DOI:** 10.3389/fpls.2017.02261

**Published:** 2018-01-19

**Authors:** Javier Polo, Pedro Mata

**Affiliations:** ^1^R&D Department, APC Europe S.L., Granollers, Spain; ^2^Universidad Autónoma del Estado de Morelos, Cuautla, Mexico

**Keywords:** biostimulant, animal protein origin, enzyme hydrolyzed, seaweed, tomato

## Abstract

The objectives of this experiment were to determine the effects of different application rates of an enzyme hydrolyzed animal protein biostimulant (Pepton) compared to a standard application rate of a biostimulant derived from seaweed extract (Acadian) on plant growth parameters and yield of gold cherry tomatoes (*Solanum lycopersicum* L.). Biostimulant treatments were applied starting at 15 days after transplant and every 2 weeks thereafter for a total of 5 applications. One treatment group received no biostimulant (Control). Three treatment groups (Pepton-2, Pepton-3, Pepton-4) received Pepton at different application rates equivalent to 2, 3, or 4 kg/ha applied by foliar (first 2 applications) and by irrigation (last 3 applications). Another treatment group (Acadian) received Acadian at 1.5 L/ha by irrigation for all five applications. All groups received the regular fertilizer application for this crop at transplantation, flowering, and fruiting periods. There were four plots per treatment group. Each plot had a surface area of 21 m^2^ that consisted of two rows that were 7 m long and 1.5 m wide. Plant height, stem diameter, distance from head to bouquet flowering, fruit set distance between the entire cluster and cluster flowering fruit set, leaf length, and number of leaves per plant was recorded for 20 plants (5 plants per plot) at 56 and 61 days after the first application. Root length and diameter of cherry tomatoes were determined at harvest from 20 randomly selected plants. Harvesting yield per plot was registered and production per hectare was calculated. Both biostimulants improved (*P* < 0.05) all vegetative parameters compared with the control group. There was a positive linear (*P* < 0.001) effect of Pepton application rate for all parameters. The calculated yield was 7.8 and 1 Ton/ha greater that represent 27 and 2.9% higher production for Pepton applied at 4 kg/ha compared to the control and to Acadian, respectively. In conclusion, Pepton was effective improving yield of gold cherry tomatoes under the low stress ambient growing conditions of this experiment. Probably short-chain peptides present in Pepton are involved in endogenous hormones and metabolic mediators that could explain the results obtained in this study.

## Introduction

Biostimulants have been defined as substances, microorganisms or materials, excluding nutrients and pesticides, which have the capacity to beneficially modify plant growth (Saa et al., 2015). The European Biostimulant Industry Council^1^ stated that “*plant biostimulants stimulate natural processes to enhance/benefit nutrient uptake, nutrient efficiency, tolerance to abiotic stress, and crop quality*.” Use of biostimulants has grown dramatically over the past decade and it is expected to achieve US$2 billion by the year 2018 (Saa-Silva et al., 2013; Calvo et al., 2014).

The theoretical effect of these proposed modes of action would be that less nutrient demand would be required to support non-productive stress related functions so that more nutrients could be used for support of productive functions, such as plant growth (Brown and Saa, 2015). However, the exact knowledge about the mode of action of biostimulants is lacking. Biostimulants have been proposed to interact with the signaling process to help the plant more efficiently respond to stress and/or to stimulate growth of bacteria, yeast, and fungi to produce molecules that benefit the plant.

Biostimulants are derived from a wide variety of different biological and inorganic materials, humic and fulvic substances, microbial fermentation of either animal or plant feedstocks, macro and micro-algae, protein hydrolysate from both vegetables and animal derivatives, industrial wastes, etc. Biostimulants are prepared using very divergent manufacturing processes. Biostimulants can be classified by source of origin (seaweed, vegetal, or animal) and content (nutritional composition) into three major groups; humic substances, seaweed extracts, and products containing amino acids and peptides (Kauffman et al., 2007). Protein hydrolysates increase nutrient uptake, especially nitrogen and iron, due to increased enzyme activity (Cerdán et al., 2009). Protein hydrolysates also have chelating activities that reduce the impact of different stressors on plant growth (Colla et al., 2014). Hydrolyzed protein may contain a variety of bioactive peptides with low molecular weight, generally known as short-chain peptides, that have been proposed to have hormonal and immunological-like activities (Ito et al., 2006; Kondo et al., 2006; Phelan et al., 2009; Colla et al., 2014; Lachhab et al., 2014). Short-chain peptides are usually considered to have <50 amino acids.

Biostimulants derived from seaweed are commonly used in the agriculture industry. The composition of seaweed biostimulants typically includes macro- and micro nutrients, free amino acids, sugars, vitamins, cytokinins, auxins, abscisic acid (ABA)-like growth substances, and betaine, which are known to impact stress signaling and response molecules (Khan et al., 2009; Minocha et al., 2014; Saa et al., 2015). Seaweed provides positive effects on growth parameters and biomass of different crops either under stress or non-stress conditions (Saa et al., 2015). These beneficial effects have led to the speculation that seaweeds extract action implies the presence of more than one group of plant-promoting substances/hormones.

Pepton 85/16® (Pepton) is a biostimulant product of natural origin that is available in micro-granular form, highly soluble in water, and produced using a proprietary enzymatic hydrolysis of animal protein (APC Europe S.L., Spain). Pepton contains high amounts of L-α amino acids (84.83%), free amino acids (16.52%), and organic-nitrogen content (12%) with low mineral-nitrogen composition (1.4%), medium potassium (4.45%), and high iron (4061 ppm) content.

Acadian Suelo (Acadian) is a balanced foliar biostimulant based on seaweed extracts (*Ascophyllum nodosum*) that are suitable to meet the requirements of high-yield crops in field, greenhouse and hydroponics intensive production systems. Acadian Suelo contains 0.34% Nitrogen, 6.84% potassium, 14.16% organic matter, 64.5 ppm boron, and 40 ppm iron.

Tomato is considered one of the most commercially vegetable crop in the world and is moderately tolerant to various abiotic stress. Plant growth and development and yield potential of crop are affected/limited by several factors like drought, salinity and extreme temperatures (Srinivasa Rao et al., 2016). Heat stress is the major constraint in tomato cultivation (Abdul-Baki, 1991). Optimal temperature for tomato is between 20 and 25°C. Temperatures over 35°C will affect the germination, flowering, fruit set, and fruit ripening in tomato (Srinivasa Rao et al., 2016). Chilling is also seriously affecting tomato growth and development. Low temperature affects plants by causing cells and tissues dehydration by crystallization of the cellular water (Pearce, 2001). In addition, other stress situation like salt stress and water stress affects plant growth, yield, and fruit quality (Srinivasa Rao et al., 2016).

The objectives for this study were to determine the effectiveness of biostimulants (Acadian or Pepton) on yield and growth parameters for cherry tomatoes grown under low stress ambient field plot conditions and to determine if different application rates of Pepton had a linear effect on the measured parameters. Furthermore, as the mode of action of seaweed extract is better known, and even though the investigation of the mode of action of the Pepton product is beyond the scope of this manuscript, we will try to speculate on possible similarities in the way of action of both products to explain the results obtained.

## Materials and methods

### Biostimulants tested

Two biostimulant products were tested: a product derived from enzymatically hydrolyzed animal protein, known commercially as PEPTON 85/16® (Pepton; manufactured by APC Europe, S.L., Spain) and Acadian Suelo produced from seaweed plant extracts (Acadian; manufactured by Acadian Seaplants Ltd., Canada). Chemical and amino acid composition of these biostimulants are presented in Table 1.

**Table 1:** Typical chemical and amino acid composition of Pepton and Acadian Suelo as reported by the manufacturers.

	**Pepton**	**Acadian suelo**
**CHEMICAL COMPOSITION**		
Total organic matter, %	79.0	14.2
Total nitrogen, %	13.0	0.34
Organic nitrogen, %	12.0	NA^a^
Ammonia nitrogen, %	1.0	NA
Ratio Carbon/Nitrogen	3.8	NA
Potassium oxide (K2O), %	4.0	6.8
Phosphorous (P2O5), %	0.3	0.3
Sulfur, ppm	NA	3,950
Calcium, ppm	300	0.17
Magnesium, ppm	500	230
Iron, ppm	3,000	40.2
Boron, ppm	NA	64.5
Manganese, ppm	NA	3.60
Zinc, ppm	NA	7.48
**AMINO ACID COMPOSITION**		
Alanine, %	6.90	0.08
Arginine, %	3.22	ND^b^
Aspartic acid, %	9.93	0.17
Cysteine	<0.1	ND
Glutamic acid, %	7.25	0.27
Glycine, %	4.06	0.06
Histidine, %	6.34	ND
Isoleucine, %	0.15	0.03
Leucine, %	10.99	0.08
Lysine, %	7.19	0.03
Methionine; %	0.71	0.02
Phenylalanine, %	5.93	0.05
Proline, %	2.84	0.06
Serine, %	3.88	ND
Threonine, %	2.47	ND
Tryptophan, %	1.25	0.01
Tyrosine, %	1.92	0.03
Valine, %	6.79	0.06
Total amino acids, %	84.83	0.95
Free amino acids, %	16.52	NA

a *NA, Data not available*;

b *ND, Not detected*.

### Crop

Gold cherry tomato variety (*Solanum lycopersicum* L.) was selected and the seeds were initially raised in greenhouse and transplanted when the plants had 20–25 cm high in soil (vertisols) plots at a commercial field farm located in Mazatepec (Morelos state, Mexico). The commercial farm was located at 18°43′37′′N, 99°21′42′′O and at 960 m altitude. The climatic conditions of this area are considered warm and humid. Vertisols soils have medium to high fertility with clay texture that when dry forms cracks and when wet becomes sticky (FAO, 2007).

### Experimental design

A complete randomized block experimental design was used with four experimental plot units (replications) per biostimulant treatment group. Each experimental plot unit had a surface area of 21 m^2^ that consisted of two rows that were 7 m long and 1.5 m wide.

There were 5 different treatment groups, one control which did not received any biostimulant, and four biostimulant groups. Three biostimulant groups received Pepton 85/16 at different doses, and the fourth Acadian Suelo. Each biostimulant was initially applied 15 days after transplantation, when plants were in vegetative growth, and thereafter every 2 weeks resulting in a total of 5 biostimulant applications during the complete study.

In Pepton groups, the two first applications were foliar at 150, 225, and 300 g Pepton/100 L water, respectively, in groups Pepton-2, Pepton-3, and Pepton-4, and the three remaining doses of Pepton were applied by irrigation at 2, 3, and 4 kg/ha, respectively. Group Acadian was irrigated at 1.5 L/ha for all 5 applications following the recommendation of the manufacturer.

All groups received the regular fertilizer for this crop (328N-85P2O5-57K2O) at transplantation, flowering, and fruiting periods.

Actara® (Syngenta, Switzerland) was applied at the start of vegetative growth and 45 days later to all plots at 200 g/ha to control the effect of silverleaf whitefly (*Bemisia tabaci* Gen). Cabrio C® (BASF, Germany) was applied at the initial flowering and every 21 days thereafter (3 times) at 0.8 Kg/ha to control blight of tomatoes (*Alternaria* solani). These two specific products were applied to all experimental treatments.

### Plant growth and performance measurements

Measurements were done on 5 randomly selected plants per experimental unit (20 plants per treatment). Plant height, stem diameter, distance from head to bouquet flowering, fruit set distance between the entire cluster and cluster flowering fruit set, leaf length, and number of leaves per plant was recorded at 56 and 61 days after the first biostimulant application. Root length was determined only at harvest for 5 plants per experimental unit (20 plants per treatment).

The diameter of 5 cherry tomatoes per experimental unit was measured and recorded (20 fruits per treatment).

The total production per experimental unit (E.U.) was registered and this amount was extrapolated to the production per hectare (tons/ha) to determine harvest yield.

The scale proposed by the European Weed Research Society (EWRS) was used for assessment of phytotoxicity scoring and the results were translated to the percentage scale. According to this scale, provides values from 1 (absence of observed phytotoxicity) to 9 (complete death) (López-Nicora and Salas-Pino, 2013).

### Statistical analysis

For all parameters, ANOVA test were conducted using the software package SPSS 10 for Windows (SAS Inc., Cary, NC, USA). Significance (*P* ≤ 0.05) was identified by the General Linear Model (GLM) procedure with the Tukey mean comparison test. Effects of Pepton application rate for 2, 3, or 4 kg/ha with control group serving as 0 kg/ha were evaluated using linear, quadratic and cubic contrasts with *P* < 0.05 considered significant.

## Results

During the time of the study, the average temperature in the Morelos state (Mexico) was 20.0 ± 0.9°C (max. 27.8 ± 0.2°C and min 12.2 ± 1.6°C). Relative humidity was 55 ± 9% (min 38% and max 78%). The average rainfall was 24.2 mm. This climatology was considered within the normal values for this state (Conagua, 2013, 2014).

The vegetative results obtained in this study are provided in Table 2. Root length at harvest was increased with the application of Pepton and Acadian (*P* < 0.05). There was an increasing linear effect of Pepton application rate for root length (*P* < 0.001). Acadian results (22.27 cm) was similar to the root length observed with the application of Pepton-2 inclusion level (22.14 cm) and lower (*P* < 0.05) than values observed for the application of 4 kg/ha of Pepton (Pepton-4).

**Table 2:** Average vegetative parameters for cherry tomatoes (*Solanum lycopersicum* L.) subjected to Pepton at different application rates or Acadian compared to a non-biostimulant control.

**Parameter**	**Biostimulant treatment group^1^**					**Pepton application rate^2^**		
	**Control**	**Pepton-2**	**Pepton-3**	**Pepton-4**	**Acadian**	**SEM**	**Linear (*P* =)**	**Quadratic (*P* =)**	**Cubic (*P* =)**
Root length (cm)	20.11^c^	22.14^b^	22.47^b^	23.46^a^	22.27^b^	0.227	<0.001	0.453	0.184
**PLANT HEIGHT (m)**									
Evaluation 1^3^	1.69^c^	1.72^b^	1.73^ab^	1.75^a^	1.74^ab^	0.008	<0.001	0.888	0.716
Evaluation 2^3^	1.76^d^	1.79^bc^	1.80^ab^	1.82^a^	1.81^ab^	0.007	<0.001	0.811	0.516
**STEM DIAMETER (cm)**									
Evaluation 1	1.13^c^	1.25^b^	1.32^a^	1.34^a^	1.33^a^	0.020	<0.001	0.314	0.429
Evaluation 2	1.61^d^	1.79^c^	1.86^bc^	2.09^a^	1.94^b^	0.035	<0.001	0.014	0.068
**LEAF LENGTH (cm)**									
Evaluation 1	38.54^d^	40.19^c^	41.56^b^	42.18^a^	41.10^b^	0.206	<0.001	0.924	0.115
Evaluation 2	39.93^d^	43.81^c^	44.13^bc^	44.69^a^	44.47^ab^	0.187	<0.001	0.035	0.035
**LEAVES PER PLANT (*****n*****)**									
Evaluation 1	12.70^c^	16.00^ab^	16.25^ab^	17.10^a^	15.65^b^	0.392	<0.001	0.056	0.285
Evaluation 2	14.45^c^	16.60^b^	17.55^b^	18.95^a^	18.65^a^	0.241	<0.001	0.552	0.553
**HEAD TO BOUQUET FLOWER (cm)**									
Evaluation 1	24.50^a^	22.86^b^	22.64^b^	22.82^b^	22.77^b^	0.287	<0.001	0.013	0.996
Evaluation 2	23.95^a^	20.34^b^	20.73^b^	20.49^b^	21.36^b^	0.367	<0.001	<0.001	0.107
**CLUSTER SET (cm)^4^**									
Evaluation 1	39.15^a^	37.95^ab^	37.48^b^	37.38^b^	37.00^b^	0.541	0.013	0.563	0.881
Evaluation 2	39.51^a^	36.72^b^	35.89^c^	35.16^d^	35.87^c^	0.119	<0.001	<0.001	0.544
Fruit diameter (cm)	2.39^c^	2.60^b^	2.67^b^	2.79^a^	2.67^b^	0.025	<0.001	0.957	0.407
Estimated tomato yield (kg/EU)^5^	85.5^c^	103.9^c^	106.8^b^	109.1^a^	106.0^c^	2.071	<0.001	<0.001	0.387
Estimated tomato yield (Ton/ha)^5^	28.52^d^	34.63^c^	35.61^b^	36.38^a^	35.34^b^	0.690	<0.001	<0.001	0.388

1 *Vegetative parameter values are an average of 20 plants from 4 plots (5 plants per plot) for each treatment group. Treatments were: control, no biostimulant; Pepton at 2, 3, or 4 kg/ha (Pepton-2, Pepton-3, and Pepton-4, respectively); and Acadian at 1.5 L/ha. Biostimulant application 1 was provided 15 days after transplant and the remaining applications were provided every 2 weeks thereafter*.

2 *Linear, quadratic, and cubic treatment contrasts for the effect of Pepton application rates 2, 3, and 4 kg/ha with control serving as 0 kg Pepton/ha*.

3 *First and second evaluations were done 56 and 61 days after the initial application of biostimulant*.

4 *Fruit set distance between the entire cluster and cluster flowering fruit set*.

5 *Values are an average of 4 plots for each treatment group. E.U. = Experimental unit*.

abcd *Treatment means within row with uncommon superscripts differ (P < 0.05) using the Tukey test*.

Both biostimulants increased plant height compared with the control treatment during the first (day 56) and second (day 61) evaluations. During both evaluations, there was a linear increase for plant height with increased application rate of Pepton (*P* < 0.001). Plant height for Acadian (1.81 m at d 61) was similar to Pepton-3 (3 kg/ha). A similar pattern of results for the different treatment groups were observed for stem diameter, leaf length, or number of leaves per plant. A linear increase for stem diameter, leaf length, and number of leaves per plant was observed with increased application rate of Pepton (*P* < 0.001). A quadratic effect of Pepton was observed at the second evaluation of stem diameter (*P* = 0.014) and leaf length (*P* = 0.035) and a cubic effect (*P* = 0.035) was noted for the second evaluation of leaf length. The results obtained for the Acadian treatment for these parameters was similar to the results obtained with Pepton-3 or Pepton-4 treatments.

Distance between head to bouquet flowering and fruit set distance between the entire cluster and cluster flowering fruit set were reduced with the application of both biostimulants compared with the control treatment (Table 2). There was a linear reduction (*P* < 0.001) and quadratic (*P* < 0.001) effect of increased Pepton application rate for distance between head to bouquet flowering for both evaluations. There was a linear reduction of distance between fruit clusters for increased application rate of Pepton for both evaluations and a quadratic reduction was also observed during the second evaluation. The Acadian treatment group had similar results for distance between head to bouquet flowering (21.36 cm at second evaluation) and distance between fruit clusters (35.87 cm at second evaluation) compared to the Pepton-3 treatment group (20.73 and 35.89 cm for head to bouquet flower and cluster set, respectively). However, the use of Pepton at 4 kg/Ha resulted in reduced (*P* < 0.05) distance (35.16 cm) between fruit clusters at second evaluation compared to Acadian treatment.

Both biostimulants increased fruit diameter compared to the control and there was a linear increase (*P* < 0.001) of fruit diameter as application rate of Pepton was increased. Fruit diameter was 2.39 cm for control group and 2.60, 2.67, 2.79, and 2.67 cm for Pepton-2, Pepton-3, Pepton-4, and Acadian groups, respectively.

Tomato yield per experimental unit (EU) and estimated yield per ha were increased with the addition of both biostimulants compared to the control. There was a linear (*P* < 0.001) and quadratic (*P* < 0.001) increase of yield with increased application rate of Pepton. The increase in yield observed with Acadian (106.0 kg /EU) was similar to that of Pepton-3 (106.8 kg/EU; Table 2). However, Pepton-4 (109.1 kg/EU) increased yield (*P* < 0.05) compared to Acadian.

No phytotoxicity was observed for any of the treatment groups, therefore the value of phytotoxicity according to the EWRS for both biostimulants was classified with a value of 1 which means no effect over the crop.

## Discussion

The results from the present study demonstrated that both biostimulants had clear positive effects on vegetative parameters and yield of tomatoes grown in field plots under low stress ambient conditions at the study location. However, the origin of both biostimulants was completely different; one was based on enzymatically hydrolyzed peptides of animal origin and the other on seaweed extracts. These results may suggest that both biostimulants contain some common molecules or functional compounds that activate the crop signaling mechanism to increase the use of nutrients available for productive functions. However, due to the different nutritional composition of both biostimulants tested, it may be also possible that different molecules present specifically in each biostimulant be responsible of the effect observed. Although, the NPK contribution by both biostimulants was very limited (range of 1.3–2.6 kg N, 0.03–0.06 Kg P, and 0.4–0.8 kg K for Pepton at 2–4 Kg/ha and 0.026, 0.02, and 0.4 kg for N, P, and K for Acadian, respectively) compared with the regular fertilization program, both biostimulants provide amino acids (much more in case of Pepton) and minerals (mainly iron in case of Pepton and sulfur and boron in case of Acadian) that may have some contribution to the growth improvements observed when apply these biostimulants. However, although biostimulants may contain different levels of mineral, they are unable to provide all the nutrients needed to cover the plant required quantities (Schmidt et al., 2003). Their main benefit is to improve plant mineral uptake by roots and in leaves (Mancuso et al., 2006; Vernieri et al., 2006).

How crop signaling mechanisms are achieved by either of these biostimulants is unknown and was beyond the objectives of this experiment. Several authors suggest that biostimulants increase enzyme and chelating metal activities and can produce a pseudo-hormonal effect on a variety of crops (Cerdán et al., 2009; Colla et al., 2014; Lachhab et al., 2014; Brown and Saa, 2015).

Pepton is a biostimulant with more than 16% free amino acids that can serve as readily available fuel for crops during periods of stress. Pepton contains peptides with an average molecular weight around 2,000–3,000 daltons and 100% of the peptides have a molecular weight <10,000 daltons. About 66% of the peptides in Pepton are considered short-chain amino acids (with <50 amino acids per chain). Several authors (Ito et al., 2006; Kondo et al., 2006; Phelan et al., 2009; Colla et al., 2014) indicate that short-chain peptides may have pseudo-hormonal and immune activity on plants. Approximately 16% of peptides in Pepton are long-chain peptides (>50 amino acids) that, when applied by irrigation, can serve as food for soil microorganisms and be quickly hydrolyzed into small peptides that can be used directly by the plants.

Many abiotic factors such as temperature, salinity and drought are manifested as osmotic stress and cause secondary effects like oxidative stress. These are known to damage DNA, lipids, carbohydrates, and proteins, and in addition, cause abnormal cell signaling (Arora et al., 2002). Past research has demonstrated that Pepton to dramatically reduces the effects of thermal stress on plants. Pepton was applied to lettuce plants that were subjected to short-term episodes of either intense cold stress or heat stress (Polo et al., 2006). After these thermal episodes, different application rates of Pepton were applied in the lettuce plant growth medium. Biometric measurements were done on the lettuce plants several days after being subjected to the episodes of either cold or heat stress. Pepton treatment greatly reduced the harmful effects caused by intense cold or heat episodes. At the highest inclusion level tested, Pepton completely reversed the negative impact of the cold or heat induced thermal stress. Another study conducted with strawberry plants stressed by being transplanted and subjected to conditions of intense cold ambient temperatures showed that Pepton application increased biomass of newly formed roots, early flowering, and early production of fruit (Marfà et al., 2009). Likewise, seaweed extracts have demonstrated that alleviate abiotic stress in crop plants. Seaweed extracts from *A. nodosum* have been shown in grape wines to reduce leaf osmotic potential, a key indicator of osmotic tolerance (Wilson, 2001). In addition, a number of studies suggest that the beneficial anti-stress effects of seaweed extracts may be related to the cytokinin activity and incinerated seaweed extracts showed reduce effectiveness suggesting the organic nature of the bioactive compound (Zhang and Ervin, 2004). Cytokinins mitigate the stress-induced by free radicals by direct scavenging and preventing reactive oxygen species (ROS) formation by inhibiting xanthine oxidation. Although the presence of cytokinins in Pepton is unknown, the results observed applying Pepton under abiotic stress situations suggest that the mechanism of action could imply regulation of the osmotic tolerance and reduction of oxidative stress. Peptides change osmotic potential and some authors (Suetsuna et al., 2000; Moure et al., 2006) have reported that small-size peptides (<1 kDa) contains the best antioxidant properties by neutralizing the oxidative radical that is damaging the cells. Pepton contains free amino acids and small peptides that can be responsible for the benefits observed when it is apply under abiotic stress.

Hydrolyzed proteins applied to roots have been reported to increase nitrogen assimilation through an increase of nitrate reductase and glutamine synthetase activity (Ertani et al., 2009). In the current study with tomatoes, Pepton linearly increased the root length with increased application rate and a 16.6% longer root was achieved with the application of 4 kg Pepton/ha. Increased root length may help the plant to increase nutrient absorption and therefore improve the vegetative growth, as observed in this study. A previous study conducted with Pepton application to strawberry plants resulted in 1.74 higher ratio between dry weights of new roots to total roots compared to the control treatment (Marfà et al., 2009). A similar observation has been indicated for other biostimulants including plant-derived protein hydrolysates (Colla et al., 2014) and seaweed extracts (Khan et al., 2009). In general, biostimulants enhance root development by both, improving lateral formation (Vernieri et al., 2005) and increasing total volume of the root system (Mancuso et al., 2006). Several authors suggested that biostimulant induced root growth, stimulated fruit development, and growth of the flowering parts were due to auxin and gibberellin-like activities which are phyto-hormones involved in the regulation of vegetal growth (Cohen and Bandurski, 1982; Philipson, 1985; Parrado et al., 2008). Seaweed extract has been also reported to improve root development (Khan et al., 2009). Seaweed applications reduce transplant shock in tomato by increasing root size and vigor (Crouch and van Staden, 1992). This improvement could be influenced by endogenous production of auxins in the planta as well as other components present in the extract, but the stimulatory activity was lost when incinerate suggesting that the active principles were organic in nature (Finnie and van Staden, 1985). Although it would be very unlikely to find vegetables hormones, including auxin in an enzymatically hydrolyzed animal protein product as Pepton, it has been suggested that short chain peptides could act as signaling substance that may stimulate the endogenous production of auxin (Colla et al., 2014). In addition, precursors for the synthesis of auxin are aromatic amino acids as tryptophan and phenylalanine (Dai et al., 2013; Kincses and Kovács, 2013; Zhao, 2014). Furthermore, Hess (1975) stated that the biosynthesis of cinamic acids (which are the starting materials for the synthesis of phenols) are derived from phenylaniline and tyrosine. Tyrosine is hydroxyl phenol amino acids that is used to build neurotransmitters and hormones (Soad et al., 2010). Pepton contains high levels of Phe (5.93%) and moderate levels of Trp (1.25%) and Tyr (1.92%) that may explain some of the results on root development, vegetative growth, and flowering observed in our study.

Our data on growth performance are similar to other publications reporting increased plant height and number of flowers when tomato plants were treated with a protein hydrolysate product (Siapton), although not improvement was observed in the number of fruits per plant after 18 weeks growth in warehouse (Parrado et al., 2008). Colla et al. (2014) applying a plant-derived protein hydrolysate in tomato plant observed enhanced growth by +19.5% in shoot, +27.5% in root, and +20.5% for total dry biomass that is similar to what we observed in our study. Likewise, Soad et al. (2010) observed in strawflower a significant promotion for all growth and flowering parameters with increasing concentration of Pepton from 250 to 1000 ppm applied by foliar spraying, furthermore, the authors also observed an increase in the contents of chlorophyll a and b in comparison to those of untreated seedlings. In addition, seaweed extracts have shown in different studies that foliar application leads to enhanced root development and biomass accumulation in different plant varieties including tomato (Crouch and van Staden, 1992; Calvo et al., 2014) in a similar percentage that observed in our study. Also, as found in our trial, seaweed extract has been reported to trigger flowering and fruit set in several crop plants, including tomato (Crouch and van Staden, 1992; Khan et al., 2009) and to improve fruit yield but also large size fruits with superior quality (Crouch and van Staden, 1992; Khan et al., 2009). Therefore, our results improving tomato growth parameters are in the same range of improvement to other studies reported in the literature when using seaweed concentrates and hydrolyzed proteins from both, animal or vegetal origins. Seaweed extracts from *A. nodosum* contain betaines and the enhanced leaf chlorophyll content in plants treated with this seaweed extract might depend on its betaine contains (Blunden et al., 1997). Unfortunately, in the present study, the chlorophyll content was not measured in the different treatments, although comments from researchers managing the study reported that the biostimulant-treated plants were greener than controls. It is unlikely that Pepton could contains natural betaine in its composition, therefore the positive effect on green color observed in this and other studies can be mediated by metabolites involved in the endogenous betaine production of the plant. In addition, glutamic acid, glycine, and in less extend alanine and arginine are fundamental metabolites in the process of chlorophyll synthesis (Von Wettstein et al., 1995). Pepton contains significant levels of these amino acids (7.25, 4.06, 6.90, and 3.22% for Glu, Gly, Ala, and Arg, respectively) that may explain the greener color usually observed with the application of this product.

The estimated cherry tomato yield for the Pepton-4 treatment was calculated to be 7.8 Ton/ha greater than the control treatment (27% more) under the low stress ambient field conditions of this study. The improved yield generated by the Pepton-4 treatment has a significant value for the producer and could potentially be of much greater value during worst stress situations, especially in case of extreme heat stress that negatively impact yield and fruit quality (Abdul-Baki, 1991). Yield increases in seaweed treated plants are believed to be associated to hormonal substances present in the extracts, especially cytokinins that are in higher concentrations in tomato fruits compare with untreated fruit (Featonby-Smith and van Staden, 1984).

## Conclusions and future perspectives

In conclusion, Pepton was effective improving the growth and yield of gold cherry tomatoes under low stress ambient growing conditions. However, although we speculated in the present manuscript about potential modes of action of Pepton, the mechanism(s) of actions of Pepton-elicited physiological response is largely unknown. Future studies must be developed to determine mechanisms of action and identify components in Pepton that are responsible for the effects observed. Furthermore, presently the available technology allows to know the complete genome of a wide number of plants, therefore it is possible to look at the effect of Pepton on the whole genome/transcriptome to better understand the mode of action of this animal protein hydrolyzed product. According to the Intergovernmental panel on climate change (IPCC) it is likely that climate change is responsible for present and future challenges in the food production due to the increasing occurrence of abiotic stress that potentially will reduce yields and/or have an impact on crops during this century (IPCC, 2007), therefore research oriented to the development of safe biostimulants that are environmentally friendly to alleviate these stresses should be a priority.

## Author contributions

JP: Designed the experiments, reviewed the data and prepared the manuscript; PM: Conducted the experiments, analyzed data, and reviewed the manuscript; Both authors have read the manuscript and agreed with its content.

### Conflict of interest statement

Funding for this study was provided by APC Europe, S.L. but did not have any additional role in the study design, data collection and analysis, decision to publish, or preparation of the manuscript. The authors have read the journal's policy and the authors of this manuscript have the following competing interests: JP is employed by APC Europe, S.L. This does not alter our adherence to all the Frontiers in Plant Science policies on sharing data and materials. APC Europe, S.L. manufacture and sells Pepton 85/16 one of the biostimulants used in this experiment. The other author declares that the research was conducted in the absence of any commercial or financial relationships that could be construed as a potential conflict of interest.

## Footnotes

^1^www.biostimulants.eu
